# Stroke survivors’ health literacy is not associated with caregiver burden: a cross-sectional study

**DOI:** 10.1038/s41598-025-89523-x

**Published:** 2025-02-08

**Authors:** Andrea Hess Engström, Sebastian Lindblom, Maria Flink, Susanna Söderberg, Lena von Koch, Charlotte Ytterberg

**Affiliations:** 1 Department of Neurobiology, Care Sciences and Society, Karolinska Institutet, Stockholm, Sweden; 2 Women’s Health and Allied Health Professionals Theme, Karolinska University Hospital, Stockholm, Sweden; 3 Research and Development Unit for Older Persons, Region Stockholm, Sweden; 4 Theme of Heart & Vascular and Neuro, Karolinska University Hospital, Stockholm, Sweden

**Keywords:** Caregivers, Care recipient, Self-management behaviours, Care transitions, Cross-sectional, Functioning, Health care, Neurology, Risk factors

## Abstract

Caregivers to stroke survivors often experience a multifaceted strain defined as caregiver burden. Low health literacy among caregivers may contribute to increased caregiver burden but there is limited research specifically examining the association between stroke survivors’ health literacy and caregiver burden. Therefore, the aim here is to explore if there is an association between stroke survivors’ health literacy and caregiver burden one year after stroke. Participants were 50 caregivers and 50 stroke survivors who were followed up in a longitudinal study on care transitions after stroke. Data were collected one year after the stroke survivors’ discharge from hospital and analysed using ordinal logistic regression. Most of the caregivers, median age 71 years, reported being satisfied with their lives (85%) and a low caregiver burden (74%). Stroke survivors’ health literacy was not associated with caregiver burden. However, lower needs of assistance in daily activities, lower levels of depression, higher levels of participation and increased age in stroke survivors were associated with lower caregiver burden. In conclusion, stroke survivors’ health literacy was not associated with caregiver burden one year after stroke. Future studies with larger samples, focusing on populations with lower functioning after stroke and higher caregiver burden, are recommended.

## Introduction

Stroke is the second leading cause of death worldwide, with incidence increasing with age^1,2^. Long-term consequences after stroke, such as sensorimotor and cognitive disabilities^1^, are common, and these require rehabilitation and ongoing support from caregivers such as family members^3^.

Being a caregiver has been described as a full-time job and may impact various aspects of the caregivers’ life such as their own well-being, participation in activities, and the relationship with the care recipient^4^. Caregivers may experience multifaceted strain defined as caregiver burden^5^, significantly impacting their health and well-being^6^. The prevalence of caregivers experiencing a significant burden ranges from 25 to 54%, with some studies suggesting variation over time^7^. A previous study found that 32% experience a high caregiver burden at some point in the first year after the care recipient’s stroke, and high caregiver burden is associated with living together with the stroke survivor^8^.

Both factors associated with the caregiver and factors associated with the stroke survivor may increase caregiver burden. Factors related to the caregiver include spending more time on caregiving, experiencing mental health problems, reporting unmet needs, and having low life satisfaction^9–11^. Low levels of participation among stroke survivors are also associated with increased caregiver burden^12,13^. Further, higher caregiver burden can in turn contribute to higher anxiety and depression symptoms, as well as a poorer sense of coherence in caregivers^9,14^. There are conflicting results in the literature regarding the care recipient’s age and its impact on caregiver burden^11^. However, evidence seems consistent regarding the association between higher caregiver burden and care recipients’ low levels of global functioning; higher dependency in activities of daily living; impaired motor function; poor neuropsychological status and cognitive functioning; and mental health issues^11,14–16^.

Health literacy refers to an individual’s ability to “gain access to, understand and use information in ways which promote and maintain good health”, enabling effective engagement and informed health decision-making^17,18^. It is associated with better function and overall health among stroke survivors^19,20^. For people with Parkinson’s disease, low health literacy was associated with higher caregiver burden when not taking disease severity and cognitive impairment into account^21^. In other populations, the association between health literacy among caregivers and caregiver burden has been investigated. A systematic review indicated that low health literacy among caregivers may contribute to increased caregiver burden and poorer care recipient self-management behaviours^22^, and interventions increasing health literacy can reduce caregiver burden^23^.

While the relationship between caregivers’ health literacy and caregiver burden has been explored^22^, we have found no research specifically examining the association between stroke survivors’ health literacy and caregiver burden. This association is important to study as it is plausible that stroke survivors with high literacy are less dependent on their caregivers in managing their health. This knowledge could contribute to the development of targeted interventions aimed at reducing caregiver burden and enhancing the overall well-being of stroke survivors and their caregivers.

## Aim

The aim of the study was to explore if there is an association between stroke survivors’ health literacy and caregiver burden one year after stroke.

## Methods

### Study design

The study had a cross-sectional design and used an explorative approach.

### Study participants

A longitudinal study investigating care transitions in stroke survivors was carried out between 2016 and 2018. Detailed information on recruitment and data collection has previously been described^24,25^. In brief, patients with a suspected diagnosis of stroke who were referred to neurorehabilitation teams in primary care after discharge from one of two participating hospitals in the Stockholm region and their significant others were included in the study. Patients were recruited by members of the rehabilitation teams working at participating hospitals. Significant others of the included patients were also invited to participate. All participants received oral and written information about the study. Inclusion took place after a signed informed consent was obtained. Participants in the present study were those who participated in the one-year follow-up and completed the European Health Literacy Survey Questionnaire (stroke survivors) and the Caregiver Burden Scale (their significant others, hereafter named caregivers).

The study was approved by the Regional Ethics Committee in Stockholm (registration number 2015/1923–31/2) and is reported according to the Strengthening the Reporting of Observational studies in Epidemiology (STROBE) guidelines. All research was performed in accordance with the Declaration of Helsinki.

### Data collection

Data were collected from stroke survivors and caregivers. Sociodemographic data from stroke survivors were retrieved from medical records, questionnaires, and from caregivers through questionnaires. Data included age, sex, cohabiting status (yes/no) and relationship between stroke survivor and caregivers. Further, from stroke survivors, data on educational level (elementary, secondary, university/college), and working status (yes/no) were collected. From caregivers, data on assisting the stroke survivor with personal activities of daily living (P-ADL) (yes/no), and with instrumental activities of daily living (I-ADL) (yes/no), were collected.

A single item of The *Life Satisfaction Questionnaire* was used to assess overall life satisfaction in caregivers^26^. The scale ranges from 1 (very unsatisfied) to 6 (very satisfied)^27^. In the analysis, the scale was dichotomised into not satisfied (1–4) and satisfied (5–6).

Cognitive impairment among the stroke survivors was assessed using the *Montreal Cognitive Assessment* (MoCA). Lower scores (< 26 points of max 30) indicate cognitive impairment^28^.

#### Outcome variable

Caregiver burden was assessed with the *Caregiver Burden Scale*. The questionnaire consists of 22 items regarding perceived burden as a caregiver to a person with disability^29^. The items can be rated from 1 to 4 (not at all, seldom, sometimes, often), and cover aspects such as caregivers’ health, psychological well-being, relations and social network, physical workload, and aspects related to environment. The severity of the burden was classified according to the mean scores as follows: 1.00-1.99 (low burden), 2.00-2.99 (moderate burden), and 3.00-3.99 (high burden).

#### Independent variables related to stroke survivors

Stroke survivors’ health literacy was assessed using the *European Health Literacy Survey Questionnaire*^30^. It consists of 16 items and comprises four dimensions of health literacy: the ability to access, understand, process, and apply health information^31^. The total score ranges from 0 to 16 and is categorised as inadequate health literacy (< 8 points), problematic (9—12 points), and sufficient (above 13 points)^31^.

General self-efficacy was assessed using the *General self-efficacy scale*. It consists of 10 items with a total score ranging from 10 to 40^32^. Higher scores indicate higher self-efficacy^32^.

Need of assistance in ADL was assessed using the *Barthel Index* (BI)^33,34^. It consists of 10 items related to personal care and mobility with a total score ranging from 0 to 100. Higher scores indicate greater independence^34^.

Depression symptoms were assessed using the subscale Depression of the *Hospital Anxiety and Depression Scale*. The subscale Depression consists of 7 items. The total score ranges from 0 to 21. Higher scores indicate greater depression symptoms, and a score of *≥* 4 is considered a cut-off for depression symptoms after stroke^35^.

Perceived impact of stroke on participation was assessed with the *Stroke Impact Scale*,* domain participation.* The total score ranges from 0 to 100. Higher scores indicate lower perceived impact^36^.

### Statistical analysis

Descriptive statistics were used to describe caregivers’ characteristics and caregiver burden. The selected variables were based on a thorough review of the literature concerning factors associated with caregiver burden and aimed to incorporate those considered potentially influential. The explanatory variables of interest include general self-efficacy, need for assistance in ADLs, depression symptoms, and perceived participation. Covariates include the stroke survivor’s age and cohabiting status to help control for demographic variability that could influence the relationship between health literacy and caregiver burden. Due to the sample size, each model was limited to a maximum of five variables to maintain statistical validity and avoid overfitting. Therefore, variables were analyzed across different models, ensuring comprehensive coverage without compromising the clarity of the results. In a first step, univariate ordinal logistic regression was carried out to analyse single variables that were associated with caregiver burden. The variables analysed in the univariate analysis were *cohabiting status and stroke survivors’ age*,* sex*,* health literacy*,* general self-efficacy*,* need of assistance in ADL*,* depression symptoms*,* and perceived participation.* In a second step, statistical models were built to analyse the association between stroke survivors’ health literacy and caregiver burden:

- Model A: health literacy, general self-efficacy, cohabiting status, need of assistance in ADL.
- Model B: health literacy, general self-efficacy, cohabiting status, need of assistance in ADL, depression symptoms.
- Model C: health literacy, general self-efficacy, cohabiting status, need of assistance in ADL, perceived participation.
- Model D: health literacy, general self-efficacy, cohabiting status, need of assistance in ADL, stroke survivor’s age.

Caregiver burden was analysed in the regression models as an ordinal variable categorised in low, moderate, and high burden. The variables cohabiting status and sex was analysed as a dichotomous variable. The other variables used in the models were analysed as continuous variables.

Results were considered statistically significant if *p* < 0.05. All analyses were carried out with R, version 4.2.2.

## Results

Among the stroke survivors, median age was 75 and 80% were male. More than a half of the stroke survivors had a university education, and 24% were working. Health literacy was sufficient among 60%. Median general self-efficacy was 31 (IQR = 28—34), and 40% (*n* = 18) had cognitive impairment. Most of the stroke survivors were independent regarding need of assistance in ADL (78%), and 74% had no symptoms of depression. Further, median age among caregivers was 71 years, and most were cohabiting with the stroke survivors. The majority of the caregivers were satisfied with life as a whole and reported low caregiver burden. Table 1 presents the caregivers’ and stroke survivors’ characteristics.

**Table 1:** Caregivers’ and stroke survivors’ characteristics.

Caregivers *n* = 50	
Age at inclusion, median (IQR)	71 (64—76)
Sex, n (%)	
Male	11 (22)
Female	39 (78)
Relation to the stroke survivor, n (%)	
Partner (cohabiting)	44 (88)
Partner (non-cohabiting)	2 (4)
Children	4 (8)
Life Satisfaction, n (%)	
Not satisfied	7 (14)
Satisfied	41 (85)
Assisting with P-ADL, n (%)	6 (12)
Assisting with I-ADL, n (%)	23 (46)
Caregiver burden, n (%)	
Low burden	37 (74)
Moderate burden	9 (18)
High burden	4 (8)

Stroke survivors *n* = 50	
Age at inclusion, median (IQR)	75 (66–80)
Sex, n (%)	
Male	40 (80)
Female	10 (20)
Educational level, n (%)	
Elementary school	13 (26)
Secondary school	10 (20)
University/college	27 (54)
Working status, n (%)	
Yes	12 (24)
No	38 (76)
Health literacy, n (%)	
Sufficient	30 (60)
Problematic	14 (28)
Inadequate	6 (12)
General self-efficacy, median (IQR)	31 (28–34)
Need of assistance in ADL, n (%)	
Yes (BI = 0–95)	11 (22)
No (BI = 100)	39 (78)
Perceived participation, median (IQR)	90 (76–100)
Cognitive impairment, n (%)	
Yes	18 (40)
No	27 (60)

Stroke survivors’ health literacy was not associated with caregiver burden in the univariate analysis (Table 2). Other variables were found to be associated with lower caregiver burden: higher levels of general self-efficacy and perceived participation, lower levels of depression symptoms, and less need of assistance with ADL.

**Table 2:** Univariate analysis of associations between stroke survivors’ characteristics and the dependent variable caregiver burden.

Independent variables	Odds ratio	95% CI	*p*-value
Stroke survivors’ age	1.05	0.99–1.11	0.11
Stroke survivors’ sex	1.30	0.25–5.56	0.74
Cohabiting	2.43	0.43–11.9	0.29
Health literacy	1.15	0.94–1.40	0.16
General self-efficacy	1.18	1.05–1.35	**< 0.01**
Need of assistance in ADL	1.16	1.07–1.27	**< 0.01**
Depression symptoms	0.65	0.49–0.83	**< 0.01**
Participation	1.08	1.05–1.13	**< 0.001**

Stroke survivors’ health literacy was not associated with caregiver burden when controlling for different variables in the regression models (Table 3). After adjustment in the statistical models, need of assistance in ADL remained statistically significant, i.e., less need of assistance in ADL was associated with lower caregiver burden in all models except in Model C (*health literacy*,* general self-efficacy*,* cohabiting status*,* need of assistance in ADL*,* perceived participation*). Lower levels of depression symptoms, higher levels of perceived participation and increased age remained statistically significant in the models and were associated with lower caregiver burden.

**Table 3:** Regression models of associations between the dependent variable caregiver burden and the independent variable health literacy.

	Odds ratio	95% CI	*p* value
Model A			
Health literacy	0.99	0.77–1.25	0.91
General self-efficacy	1.07	0.92–1.24	0.40
Cohabiting	2.05	0.25–13.3	0.47
Need of assistance in ADL	**1.13**	**1.03–1.26**	**< 0.01**
Model B			
Health literacy	0.97	0.70–1.30	0.82
General self-efficacy	0.94	0.77–1.12	0.46
Cohabiting	0.18	0.01–2.42	0.21
Need of assistance in ADL	**1.16**	**1.05–1.30**	**< 0.01**
Depression symptoms	**0.54**	**0.32–0.82**	**< 0.01**
Model C			
Health literacy	1.00	0.77–1.31	0.99
General self-efficacy	1.01	0.84–1.19	0.93
Cohabiting	0.52	0.50–4.01	0.54
Need of assistance in ADL	1.02	0.89–1.15	0.79
Participation	**1.08**	**1.03–1.15**	**0.001**
Model D			
Health literacy	1.03	0.78–1.36	0.81
General self-efficacy	1.08	0.91–1.29	0.38
Cohabiting	4.05	0.44–34.70	0.20
Need of assistance in ADL	**1.19**	**1.07–1.36**	**< 0.001**
Stroke survivors’ age	**1.13**	**1.05–1.23**	**0.001**

## Discussion

This study aimed to explore the relationship between health literacy in stroke survivors and caregiver burden. Health literacy was not found to be associated with caregiver burden when adjusting for cohabiting status and stroke survivors’ age, general self-efficacy, need of assistance in ADL, depression symptoms, and perceived participation in the statistical models. However, less need of assistance in activities of daily living, lower levels of depression symptoms, and higher levels of participation were identified as significant contributors to a low caregiver burden.

Despite controlling for different factors, health literacy was not associated with caregiver burden. One possible explanation could be that stroke survivors’ psychological and physical abilities overshadow the importance of their health literacy. Although previous studies have predominantly focused on caregivers’ health literacy^22^, the relationship between care recipients’ health literacy and caregiver burden remains poorly understood. As the study sample consisted mostly of stroke survivors with high health literacy and a high level of independence, and caregivers with a low burden, it is conceivable that this association may manifest differently in other stroke-related samples.

Interestingly, despite the majority of caregivers living with the stroke survivor, cohabitation itself was not found to be associated with caregiver burden. The literature presents mixed findings on this topic. For instance, one study reported that higher caregiver burden was associated with not living with the stroke survivor^37^, while another found that cohabiting with the stroke survivor was associated with higher caregiver burden^8^. One possible explanation for these contradictory findings could be attributed to differences in the demographic composition of the caregiver population studied. Unlike the present study, where most caregivers were partners, the previous study involved a significant proportion of caregivers who were the children of the stroke survivors^37^.

Factors such as less need of assistance in ADL, lower levels of depression, higher levels of participation, and older age were consistently associated with lower caregiver burden across the statistical models. Our findings corroborate with a prior study where caregivers of individuals requiring greater assistance reported higher caregiver burden^14,15^, while post-stroke depression exacerbated caregiver burden^11,16^. This highlights the importance of developing strategies to support caregivers of stroke survivors with more pronounced disabilities and depression after stroke. The association between older age and caregiver burden should, however, be interpreted with caution. A previous literature review revealed inconsistent findings, suggesting that the relationship between age and caregiver burden may be influenced by other factors such as time spent on caregiving and stroke survivors’ cognitive functioning, which is not accounted for in the present study^11^.

Higher participation was associated with lower caregiver burden in the present study. An intervention programme for improving participation after acquired brain injury also reported that improved participation was associated with reduced caregiver burden^12^. This suggests that strategies to increase participation in stroke survivors may also be important to the caregivers as a way to reduce their burden.

Notably, stroke survivors’ general self-efficacy was associated with low caregiver burden only in the univariate analysis, highlighting its potential role in mitigating caregiver burden. Moreover, higher self-efficacy has been shown to be associated with lower depression symptoms and better functioning^38,39^. Self-efficacy seems to play a role in health decisions^40^ and has previously been found to be associated with higher health literacy among stroke survivors^41^. Future studies elucidating the interplay between care recipients’ self-efficacy and caregiver burden, including changes over time, are warranted.

Lastly, the sex of the caregiver was not associated with caregiver burden. Previous research has found no association between sex and caregiver burden among caregivers of persons with Parkinson’s Disease^42^, while another study found that factors associated with caregiver burden may be gender-specific among caregivers of persons with cancer^43^. How gender plays a role in caregiver burden in relation to stroke survivors is less studied and should be further explored in larger studies.

### Strengths and limitations

This study was based on a secondary analysis of data collected for a longitudinal study investigating care transitions in stroke survivors. Despite the limit sample size, the current study addresses a notable gap in the literature and provides a foundation for future research in the field. Additionally, the identification of other significant factors affecting caregiver burden can be used to tailor interventions targeting caregivers’ health. While the present study brings valuable insights into the association between stroke survivors’ health literacy and caregiver burden, the results must be interpreted with caution due to its exploratory approach and small sample size.

One limitation of this study is the exclusion of cognition impairment from the statistical models, despite evidence that cognitive functioning in individuals with brain injuries is associated with caregiver burden^11^. This decision was made due to missing data for the variable assessing cognitive impairment and concerns about potential collinearity, which could have comprised the integrity of the models.

Despite the high burden category comprising only 8% of the sample, an ordinal regression was conducted. Assuming that caregiving is a nuanced experience, this method allowed us to preserve detailed information about the distribution of caregiver burden and maintain the original categorization used in the instrument. However, this has implications for the generalizability of the results due to the sample size.

Lastly, another limitation concerns the sample of the study, with a high proportion of significant others experiencing low caregiver burden and patients with mild consequences of a stroke and high health literacy. The composition of the sample may limit generalisability to populations with high caregiver burden and more severe stroke outcomes.

## Conclusion

Stroke survivors’ health literacy was not associated with caregiver burden one year post-discharge from hospital. These results must be interpreted with caution due to the small sample size. Future studies with larger samples, focusing on populations with lower functioning after stroke, are recommended to further elucidate this association.

## Data Availability

The datasets generated during and/or analysed during the current study are available from the corresponding author on reasonable request.
